# HPLC Fingerprint Analysis with the Antioxidant and Cytotoxic Activities of Selected Lichens Combined with the Chemometric Calculations

**DOI:** 10.3390/molecules25184301

**Published:** 2020-09-19

**Authors:** Anna Hawrył, Mirosław Hawrył, Agnieszka Hajnos-Stolarz, Jagoda Abramek, Anna Bogucka-Kocka, Łukasz Komsta

**Affiliations:** 1 Department of Inorganic Chemistry, Faculty of Pharmacy, Medical University of Lublin, Chodźki 4a St., 20-093 Lublin, Poland; anna.hawryl@umlub.pl; 2 Herbapol-Lublin S.A., Diamentowa 25 Street, 20-471 Lublin, Poland; ahajnos@gmail.com; 3 Department of Biology and Genetics, Medical University, Chodźki 4A Street, 20-093 Lublin, Poland; jagoda.abramek@umlub.pl (J.A.); anna.bogucka-kocka@umlub.pl (A.B.-K.); 4 Chair and Department of Drug Chemistry, Medical University, Jaczewskiego 4 Street, 20-090 Lublin, Poland; lukasz.komsta@umlub.pl

**Keywords:** lichens, HPLC, antioxidant and cytotoxic activities, chemometrics, PLS

## Abstract

The aim of this study was to evaluate the ability of multivariate techniques to predict antioxidant and cytotoxic activity of the selected lichens from the chromatographic data. A simple and reproducible HPLC-DAD technique has been used to obtain the chromatographic fingerprint profiles. Reversed phase high performance liquid chromatography (RP-HPLC) linear gradient system with methanol, water and phosphoric acid (V) (pH 2.3) as the mobile phase was used (50 min). Principal Component Analysis (PCA) has been applied to the evaluation of the phytochemical similarity between studied samples, especially between the same species collected in various places of Poland (*Cetraria islandica* (L.) Ach., CI, *Cladina mitis* Sandst., CM, *Hypogymnia physodes* (L.) Nyl., HP). The ability to scavenge free radicals was evaluated using 2,2-diphenyl-1-picrylhydrazyl (DPPH) and ferric reducing antioxidant power (FRAP) methods and the total phenolic content was determined by Folin-Ciocalteu (F-C) test. In the case of DPPH % of inhibition was higher for selected species (*Pseudevernia furfuracea* (L.) Zopf, *H. physodes* in comparison to the literature data. The FRAP test showed that the *H. physodes* extract had higher ability to scavenge free radical in comparison to *Cladonia furcata* (Huds.) Schrader and *Evernia prunastri* (L.) Ach., whereas *P. furfuracea* extract showed higher ability than *C. islandica*. The high content of phenolics in *P. furfuracea* and *H. physodes* confirms their high antioxidant activity. The cytotoxic activity of studied extracts was tested by cell culture method using the human HL-60 / MX2 acute CKL-22 (CRL-2257) promyelocytic leukemia tumor cell line. The lowest values of IC_50_ [µg∙mL^−1^] were obtained for: *H. physodes* (HP1)—99.4; *C. digitate*—122.6; *H. physodes* (HP)—136.5, *C. subulata*—142.6; *C. mitis*—180.2.

## 1. Introduction

Lichens are defined as composite organisms, formed by the symbiotic relationship between fungus (mycobiont) and a green algae (and/or cyanobacteria) as photosynthetic partner (photobiont). They inhabit most ecosystems [1,2,3] and various specific (even extreme) living conditions of their existence, such as slow growth and long life which are the reason for the production of numerous protective compounds against various physical and biological influences [4]. Lichen primary metabolites include proteins, amino acids, carotenoids, polysaccharides, and vitamins. They are generally soluble in water and can be easily isolated from the lichens by boiling water. The secondary metabolites synthesized by lichens cover many classes of organic compounds [5]. Secondary metabolites commonly known as lichen acids have diverse biological properties such as the antimicrobial, antibacterial, cytotoxic, antioxidant, antiviral, and antifungal activities [1,2,3,4,6,7,8,9,10,11,12,13].

As literature data show, there are many papers describing the antioxidant activity of various lichens from different areas of the world, which was confirmed using 2,2-diphenyl-1-picrylhydrazyl (DPPH) reagent [14,15,16,17,18,19,20,21,22,23]. Some phenolic compounds (e.g., vesuvianic acid, usnic acid, stictic acid, lecanoric acid, sekikaic acid, lobaric acid, isidiophorin, rhizonyl alcohol, atranol, chloroatranol, hematomic acid, chlorohematommic acid) were isolated from various lichen species, such as *Lobaria pulmonaria* (L.) Hoffm., *Usnea longissima* Ach., *Parmotrema tinctorum* (Delise ex Nyl.) Hale, *Parmotrema stuppeum* (Taylor) Hale *Peltigera praetextata* (Flörke ex Sommerf.) Zopf., *Sticta nylanderiana* Zahlbr., *Lethariella canariensis* (Ach.) Krog, *Usnea articulata* (L.) Hoffm., *Lasallia pustulata* (L.) Merat., *Cetraria islandica* (L.) Ach., *Lecanora atra* (Hudson) Hafellner, *Parmelia pertusa* Schaer., *Pseudevernia furfuracea* (L.) Zopf, *Umbilicaria cylindriea* (L.) Delise, *Cetraria pinastri* (Scop.) Gray, *Cladonia digitata* (L.) Hoffm., *Cladonia fimbriata* (L.) Fr., *Fulgensia fulgens* (Sw.) Elenkin, *Ochrolechia parella* (L.) A. Massal., *Parmelia crinita* Ach., using methanol, ethanol, water, or acetone as solvents. 

The investigation of the possibility of using lichen metabolites in cancer therapy dates back to the late 1960s, when the activity of polysaccharides against cancer cells of the sarcoma line (Sarcoma-180) was studied [24,25]. For the first time, the anticancer activity of usnic acid, extracted from the genus *Cladonia* on Lewis lung, was studied and described in 1975 [26]. Since then, there has been an increase in interest and intensification of the research, both on lichen extracts and their compounds isolated on cell lines derived from various cancers [1,4,11,13,27]. Various types of in vitro cellular tests are most commonly used to determine the cytotoxic activity of the test compound. The tests are based on changes in the integrity of the cell membrane, as well as the activity of enzymes associated with cell metabolism, the ability of the cell to divide, or changes in the content of protein or genetic material.

There are many publications describing the chromatographic analysis of selected lichens using the high performance liquid chromatography, HPLC [4,8,10,13,28,29], optionally combined with mass spectrometry (HPLC-MS) and thin layer chromatography (TLC) [30], liquid chromatography-mass spectrometry (LC-MS, UPLC-MS/MS, UHPLC-QToF-MS) [31,32,33,34]. The reversed phase high performance liquid chromatography (RP-HPLC) technique with isocratic elution for the analysis of lichens extracts was used for the first time in the 1980s. Depsides and depsidones from the *Pseudevernia furfuracea* (L.) Zopf, *Evernia prunastri* (L.) Ach. and *Hypogymnia physodes* (L.) Nyl. species were separated by this technique using 13 reference substances [35]. RP-HPLC-DAD isocratic method was also applied to identification of phenolics in some lichens extracts by Gupta et al. [36]. 

The use of the gradient elution was a real milestone in the analysis of lichens: 30 min linear gradient of water-methanol + 0.5% acetic acid was used to separate extracts of the *Rhizocarpon* genus [37]. The presence of constictic, stictic, norstictic, psoromic, gyroforic, and rhizocarpic acids and seven unidentified compounds were detected by Geyer et al. [37]. In other works the gradient elution technique was also used to confirm the presence of ten compounds of *Pertusaria* species [38] or to identification of norstictic acid in extract of *Pertusaria pseudocorallina* (Lilj.) [39]. Miniaturized liquid column chromatography techniques were used by Latkowska et al. to identify of some secondary metabolites in the *Hypogymnia physodes* (L.) Nyl. [31]. Coupling liquid chromatography with spectral techniques is an effective tool for identifying unknown compounds in extracts. In this way, xanthone glycosides were separated and identified in the *Umbilicaria proboscidea* (L.) Schrader species [40], usnic acid in two species—*Usnea barbata* (L.) Weber ex F.H.Wigg. and *Xanthoparmelia chlorochroa* (Tuck.) Hale [41] by LC-MS was identified and ten compounds in the *Hypogymnia Physodes* (L.) Nyl. species were identified by UPLC-MS [31]. In other study eight metabolites (among others usnic, lechee, lobaric, and sexic acids) were quantified in eight lichen species using UHPLC-MS [32].

As is commonly known, the chromatographic fingerprint analysis is one of the methods of the preliminary chemical evaluation of herbal medicines and it has been accepted by WHO as a strategy for quality estimation of medicinal plants [42,43]. Among the literature reports, the chemical fingerprint profiles of selected lichens were created using different analytical methods: HPLC [4,10,36], direct analysis in real time mass spectrometry (DART-MS) [44], LC-MS [33], UPLC-MS/MS [34]. 

The evaluation of the complicated and extensive HPLC data is difficult and one of the right tools for this purpose are the chemometric methods. The chemometrics, as the application of mathematical and statistical techniques, is an important tool to retrieve more information from the chromatographic data and it can be used for the evaluation of the chemical (dis)similarity between the fingerprint chromatograms of selected herbal medicines [45]. These technique treats each chromatogram as unique “fingerprint” without a need of peak identification, and the measures of (dis)similarity between two samples is a quantitative coefficient representing the difference between their chemical compositions. However, before that, the raw chromatographic data should be pre-processed with the baseline correction, smoothing, normalization of signals, and alignment of chromatograms. Multivariate chemometric techniques such as Principal Component Analysis (PCA) allows to extract an analytical information from chromatograms or spectra and they have been recommended for identification and quality control of medicinal plants [46]. The combination of the chromatographic data and chemometric tools allows fast and good analysis of plant complex mixtures [47,48,49]. 

Predicting of the biological activity (antioxidant, anticancer) of plant extracts based on chromatograms (or spectra) is possible due to different multivariate calibration techniques. PLS (Partial Least Squares) is one of the most popular multivariate calibration method, based on the determination of a linear relationship between independent set of measurements X (such as a fingerprint, or peak areas) and a dependent set of properties Y (biological activity) [50,51]. The determination of the ability to scavenge free radicals by spectrophotometric techniques is time consuming. The PLS method allows to determine the regression equation into which the absorbance values (chromatographic data) can be substituted to obtain the antioxidant activity expressed by the concentration of standard (e.g., gallic acid or trolox). These calculations can be performed by a computer software, which significantly reduces the time. This technique saves a lot of time compared to the classical approaches, such as DPPH, ferric reducing antioxidant power (FRAP), and F-C (Folin-Ciocalteu) total phenolics estimation. There are some papers describing the usefulness of fingerprints as a good tool to predict the biological activity of plant material using PLS technique [52,53]. 

Based on the chromatograms obtained for individual lichens and their antioxidant and cytotoxic activity, a PLS model was constructed, which helps to predict the biological activity of each extract based on its chromatographic profile. The high value of the determination coefficient proves a good fit of the data in the model, and thus a high correlation between the chromatographic data (chromatogram) and biological properties for the analyzed plant samples was confirmed. After a proper calibration with reference method (DPPH, FRAP or F-C), the obtained model can be used to compute many activities from one chromatogram without a need to perform a classical assay for each activity.

In our study, the RP-HPLC-DAD gradient elution analysis of methanolic extracts of twenty selected lichens was performed and the presence of usnic acid was confirmed in some of them. The total phenolic content, the antioxidant (DPPH, FRAP) and cytotoxic activities of extracts were also investigated. The main aim of this paper was combination of chemical fingerprints and the antioxidant activity of lichens to establish a system using the PLS model to study active ingredients in tested plants representing its antioxidant and cytotoxic activities. To our best knowledge, few publications describe the correlation analysis between biological properties and chromatographic data in lichen analysis. The approach described in this study may be useful in controlling the quality of plant material. 

## 2. Results and Discussion

### 2.1. HPLC Results

The purpose of the present study was to evaluate the ability of multivariate techniques to predict the antioxidant and cytotoxic activities of the methanolic extracts of selected lichens (Table 1) from their fingerprint chromatograms. RP-HPLC system was used for the first time with a gradient of methanol and water acidified with phosphoric acid (V) (pH 2.3) for 50 min. 

This technique enabled the identification of (+)-usnic acid in the tested extracts. The presence of (+)-usnic acid was confirmed for the following methanolic extracts: *Cladina mitis* Sandst. (CM, CM1), *Cladina arbuscula* (Wallr.) Flotow (CSYL), *Evernia prunastri* (L.) Ach. (EP), *Hypogymnia physodes* (HP, HP1), *Xanthoria parietina* (L.) Th. Fr. (XP) and appropriate chromatograms were presented in Supplementary Material (Figures S1–S7). The presence of usnic acid in samples was confirmed based on the retention time value (retention time value was 23.978 min) and the spectrum. The concentration of usnic acid was semi-quantitatively calculated on the basis of a calibration curve determined for its nine concentrations (from 0.008957 to 0.39984, mg⋅mL^−1^). The value of the determination coefficient of the relationship between the absorbance and concentration of usnic acid was *r*^2^ = 0.99. Next, the concentrations were calculated to the dry weight of extracts and the results are presented as mg⋅g^−1^ (the abbreviations according to Table 1): CM—0.1852 ± 0.0046; CM1—0.2312 ± 0.0058; CSYL—1.8105 ± 0.0399; EP—0.3906 ± 0.0093; HP—0.1655 ± 0.0037; HP1—0.2565 ± 0.0063; XP—0.7988 ± 0.0205.

**Figure 1:**
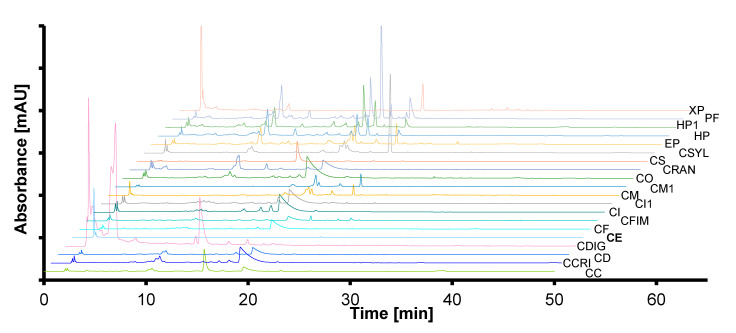
Summarized HPLC fingerprint chromatograms of lichen methanolic extracts. Symbols of lichens according to Table 1.

The comparison of chromatograms of the examined extracts in this paper can be used as a tool to confirm the identity of species that were previously botanically identified (by comparing their fingerprint chromatograms). The summarized chromatograms were presented in Figure 1. Based on the visual evaluation, the chromatograms are significantly diversified, which allows them to be used to identify the studied species using the HPLC fingerprint method with a high probability, finding the reference profile with the highest similarity (correlation, Euclidean distance etc.). The similarity of chemical composition (similar chromatographic profiles) can be useful as one of the methods of confirming the identity of the same species collected from different sites. In this work it concerns the following samples: *Cetraria islandica* (L.) Ach. (CI, CI1), *Cladina mitis* (CM, CM1), *Hypogymnia physodes* (HP, HP1). In order to confirm the phytochemical similarity between the above species of lichens (CI and CI1; CM and CM1; HP and HP1), the similarity analysis was carried out using chemometric techniques, described in the next stages in this work. 

### 2.2. Chemometric Analysis 

#### Principal Component Analysis

Various chemometric methods give the possibility of accurate, numerical comparison of chemical HPLC profiles. The literature gives a few examples of the use of chemometrics in lichen analysis [54,55,56]. The PCA analysis was used for the comparison of LC-MS *Physcia* type thallus profiles [54] or GC-MS *Cladonia* profiles [55]. The phytochemical LC-DAD-MS fingerprints of acetone extracts from *Agropsis fresiana* Müll. Arg. were also studied using the PLS technique [56]. 

**Figure 2:**
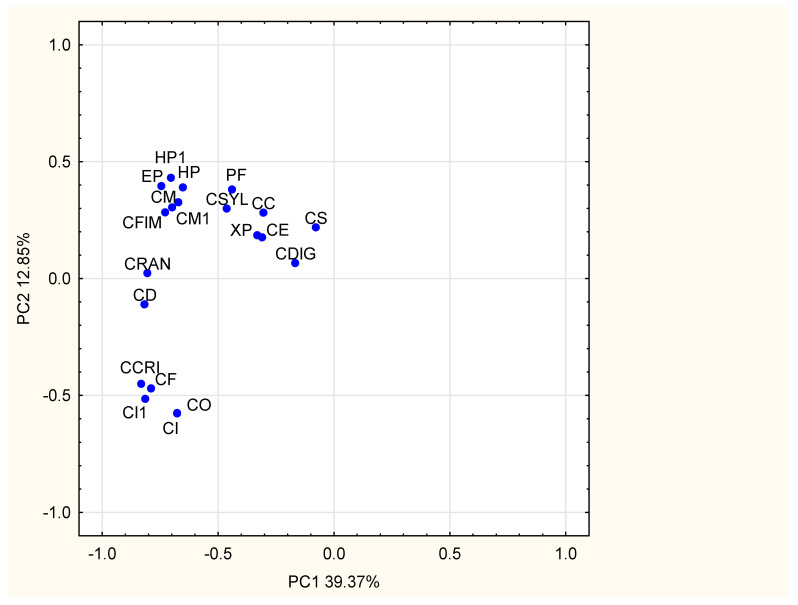
The PC1 vs. PC2 graph for methanolic lichen extracts (symbols according to Table 1).

In our study the chemical similarity between analyzed HPLC fingerprints of selected lichens extracts was also evaluated by the other chemometric tool—PCA and the PC1 vs. PC2 graph was presented in Figure 2. Close location of the samples (corresponding to lichen HPLC fingerprints, treated as objects) on the plot confirms the high similarity between them. In our study, six groups of chemically similar samples were found, points corresponding to appropriate samples are located close to each other. The first group consists of: HP, HP1, CM, CM1, EP, CFIM; the second: CRAN, CD; the third group contains: CF, CCRI, CI, CI1, CO; next group contains two samples: PF, CSYL; in the fifth group are: CE, XP, CC; and the last—CS and CDIG. The chemical similarity between lichens collected in different places (HP, CM, and CI) was confirmed.

### 2.3. Antioxidant Activity

#### 2.3.1. DPPH Test

In order to create the calibration curve of antioxidant standard (trolox) the appropriate dilutions (the data in Experimental Part) were used and the following equation was obtained: y = −10.66(±0.45)x + 0.9464(±0.01); *r*^2^ = 0.99. In order to facilitate the interpretation of results, the half maximal inhibitory concentration parameter was determined (IC_50_). It determines the concentration of antioxidant causing a decrease in the initial concentration of DPPH radical by 50%. IC_50_ was calculated as: trolox—IC_50_ = 0.0449 mg∙mL^−1^. Next, based on the calibration curves, the ability to scavenge free radicals (expressed as inhibition % and the concentration of antioxidant standards) were calculated and the results were presented in Table 2. 

The highest degree of DPPH radical scavenging was obtained for methanolic extracts of HP1, CDIG, HP, and PF with values of % inhibition 87.29, 86.72, 80.31, and 77.08, respectively, and the lowest degree of DPPH radical scavenging was found by CF extract (10.02% inhibition). As expected, the samples with the highest value of inhibition % showed the highest equivalent values of trolox (C_TE_) concentration. The same samples showed the highest antioxidant activity calculated as C_TE_ (mg∙mL^−1^): HP1 (0.0778), CDIG (0.0771), HP (0.0711), and PF (0.0690). 

As shown in literature, the different extracts of lichens were analyzed for the ability to scavenge free radicals and the results are presented by Kosanić [21,22]. The acetone, methanolic, and aqueous extracts of *Pseudevernia furfuracea* (L.) Zopf. [21] and *Hypogymnia physodes* (L.) Nyl. [22] were examined. The degree of scavenging of the DPPH radical (as inhibition %) by studied extracts from *Pseudevernia furfuraceae* (L.) Zopf. was: 87.27%, 57.88%, and 33.91% (respectively for acetone, methanolic, and water extracts) [21], and the degree of DPPH radical scavenging (as inhibition %) by acetone, methanol, and aqueous extract from *Hypogymnia physodes* (L.) Nyl. was: 60.18% inhibition, 73.18% inhibition and 30.98% inhibition, respectively [22]. In our study, the DPPH spectrophotometric results obtained for methanolic extracts of *Pseudevernia furfuraceae* (L.) Zopf. (PF), *Hypogymnia physodes* (L.) Nyl. (HP and HP1) are higher in comparison with the above cited literature data. In our experiments the % of inhibition for particular species was as follows: PF (77.08%), HP (80.31%), and HP1 (87.29%). 

#### 2.3.2. FRAP Test

In our work the antioxidant activity has been also marked by the FRAP method and expressed as the concentrations of trolox. The relationship between the absorbance and the concentration of standard was created based on the dilutions of standards presented in Materials and Methods and the calibration curve was obtained: y = 51.803(±1.40)x + 0.2017(±0.02), *r*^2^ = 0.99. Based on the above equations, the absorbances of the tested extracts were converted into concentrations expressed as trolox (C_TE_) equivalents (Table 2). 

The highest value of the ability to scavenge free radicals (calculated as trolox) was obtained for (mg∙mL^−1^): CDIG (0.057), HP1 (0.035), and HP (0.034), and the lowest—sample CSYL (0.006), according to Table 2. 

In one publication the spectrophotometric technique with FRAP was also applied to the antioxidant activity evaluation of the acetone, methanolic, and aqueous extracts of *Cladonia furcata* (Huds.) Schrad, *Hypogymnia physodes* (L.) Nyl., *Lasallia pustulata* (L.) Mérat, *Parmelia caperata* (L.) Ach. and *Parmelia sulcata* Taylor [22]. Based on this experiment, the antioxidant activity of the methanolic extracts decreased as follows: *H. physodes*, *L. pustulata*, *C. furcata*, *P. sulcata*, *P. caperata*. Similar results were obtained in our research, the sample of *H. physodes* shows stronger antioxidant activity in comparison with *C. furcata*. For *H. physodes* the concentration (mg∙mL^−1^) of trolox was equal 0.0342 (for HP) and 0.0353 (for HP1), whereas for *C. furcata* was equal only 0.0098 (Table 2). In another work, some lichen species (acetone, methanolic, and aqueous extracts) were also studied by the same FRAP technique [21]. In this study [21] the following species were analyzed: *Cetraria islandica* (L.) Ach., *Lecanora atra* (Hudson) Ach., *Parmelia pertusa* (Schrank.) Schaer., *Pseudevernia furfuraceae* (L.) Zopf. and *Umbilicaria cylindrica* (L.) Delise ex Duby. The antioxidant activity of methanolic extracts decreased in the following order for the above lichens: *L. atra*, *P. furfuraceae*, *U. cylindrica*, *C. islandica* [21]. The results from this publication [21] are partially different from ours. In general, the values obtained in our experiments are similar for PF, CI and CI1 and equal about 0.02 or 0.03 (approximately). Based on the exact trolox concentration (mg∙mL^−1^) values for these samples, it was noted that the value for *P. furfuraceae* (0.0225) was higher than one of samples of *C. islandica* (CI) which equal 0.0185, but lower than concentration value of the second sample of *C. islandica* (CI1) which equal (0.0290). Stojanović et al. [57] studied the antioxidant activity of methanolic extracts of four lichen species from the *Parmeliaceae* family: *Hypogymnia physodes* (L.) Nyl., *Evernia prunastri* (L.) Ah, *Flavoparmelia caperata* (L.) Hale and *Parmelia sulcata* Taylor. The FRAP test showed, that the antioxidant activity (expressed as concentration of ascorbic acid) of methanolic extracts was decreased as follows: *H. physodes*, *E. prunastri*, *F. caperata*, and *P. sulcata*. Similar results were obtained in our experiments, the antioxidant activity of *H. physodes* (both HP and HP1) was higher in comparison with *E. prunastri* and the values of the concentration (mg∙mL^−1^) of trolox were as follows: 0.0342 (for HP); 0.0353 (for HP1), and 0.0119 (for EP).

### 2.4. Total Phenolic Content

The total content of polyphenols was calculated with gallic acid as standard. The absorbance of gallic acid standard solutions was measured and a calibration curve was created with the equation: y = 1.6965(±0.05)x + 0.033(±0.02), *r*^2^ = 0.99. The concentrations (mg∙mL^−1^) of gallic acid were presented in Experimental Part. The total content of polyphenols in individual methanolic and methanolic-water extracts was determined on the basis of the gallic acid calibration curve (Table 2).

The highest value of total phenolic content (calculated as gallic acid concentration) was obtained for (mg∙mL^−1^): PF (1.623), CDIG (1.355), HP1 (1.317), and HP (1.264), and the lowest value for: CM1 (0.131) and CM (0.159), according to Table 2. 

As is commonly known, the ability of plant extracts to scavenge free radicals is largely associated with the presence of phenolic compounds. It has been shown that the tested extracts contain the high levels of phenolic compounds, which confirms that in this case phenolic compounds can be responsible for the antioxidant properties of lichen extracts. The highest content of polyphenols was found in methanolic extracts from the species: *Pseudevernia furfuracea* (L.) Zopf., *Cladonia digitata* (L.) Hoffm, *Hypogymnia physodes* (L.) Nyl. (HP and HP1).

### 2.5. Cytotoxic Activity

The cytotoxic activity of extracts was examined by cell culture method using the human HL-60/MX2 acute CKL-22 (CRL-2257) promyelocytic leukemia tumor cell line. IC_50_ values (concentration inhibiting the growth of cell culture by 50%) were read from the concentration of the tested extract, using plots of cell viability relationships (expressed as a percentage in relation for control). The IC_50_ values of samples were presented in Table 3. 

According to Table 3 the following extracts showed the highest ability (the lowest value of IC_50_) to inhibit the growth of cell culture (µg∙mL^−1^): HP1 (99.4), CDIG (122.6), HP (136.5), CS (142.6), CM1 (180.2). The lowest ability (the highest value of IC_50_) shows the following samples: XP (553.3), CI1 (541.8), CI (514.0), CO (511.3). 

According to the National Cancer Institute (NCI) standards of the USA, a crude extract can be considered active when IC_50_ ≤ 20 (μg·mL^−1^) [58]. Literature data indicate that usnic acid has activity against Lewis lung cancer cells [27]. Another study showed the activity of both (+)- and (−)-usnic acid against breast cancer cells (T-47D) with IC_50_ values of 4.2 and 4.0 μg∙mL^−1^, respectively and pancreatic cancer cells (Capan-2) with IC_50_ values of 5.3 and 5.0 μg∙mL^−1^, respectively [59]. (+)-usnic acid has also cytotoxic activity on tumor cell lines: L1210 IC_50_ = 6 μg∙mL^−1^, 3LL IC_50_ = 12.1 μg∙mL^−1^, DU145 IC_50_ = 15.8 μg∙mL^−1^, MCF7 IC_50_ = 17.8 μg∙mL^−1^, K-562 IC_50_ = 8.2 μg∙mL^−1^, U251 IC_50_ = 6.8 μg∙mL^−1^ [58]. In our study, the presence of usnic acid was confirmed in methanolic extracts of CM, CM1, CSYL, EP, HP, HP1 and XP (symbols according to Table 1). The obtained values of IC_50_ for these samples were low and increased as follows (cytotoxic activity decreases): HP1 (IC_50_ = 99.4), HP (IC_50_ = 136.5), CM1 (IC_50_ = 180.2), EP (IC_50_ = 355.3), CSYL (IC_50_ = 361.6), CM (IC_50_ = 418.4), XP (IC_50_ = 553.3).

Marante et al. [16] investigated the cytotoxic activity of metabolites isolated from *Lethariella canariensis* (Ach.) Krog.: atranol, chloroatranol, methyl 2,4-dihydroxy-3-formyl-6-methylbenzoate, ethyl 2,4-dihydroxy-3-formyl-6-methylbenzoate and (+)-stannic acid relative to the U937 and HL60 cell lines. IC50 values for U937 and HL60 cell lines have been shown to be 37.6 μM and > 100 μM for atranol, 8.8 μM, and 78.8 μM, respectively, for chloroatranol, 26.2 μM and 39.8 μM for methyl 2,4-dihydroxy-3-formyl-6-methylbenzoate, 74.1 μM and 100 μM for ethyl 2,4-dihydroxy-3-formyl-6-methylbenzoate, and 14.3 and 14.3 for (+)-usnic acid.

The studies of the other paper confirm, that the acetone extract from *Xanthoria parietina* (L.) Th. Fr. at a concentration of 1.5–3.0 mg∙mL^−1^ inhibits proliferation and induces apoptosis of MCF-7 and MDA-MB231 breast cancer cells [60].

Bézivin et al. [58] investigated the cytotoxic activity of hexane, ether and methanolic extracts from: *Cladonia convoluta* (Lam.) Anders, *Cladonia rangiformis* Hoffm., *Evernia prunastri* (L.) Ach, *Parmelia caperata* (L.) Ach, *Parmelia perlata* (Huds.) Ah, *Platismatia glauca* (L.) WL Culb. and C.F. Culb., *Ramalina cuspidata* (Ach.) Nyl., *Usnea rubicunda* Stirton relative to cell lines L1210, 3LL, DU145, MCF-7, K562, and U251. Hexane extracts showed the highest activity compared to the activity of the other extracts.

The cytotoxic activity of the methanolic extract from *Hypogymnia physodes* (L.) Nyl by the MTT test, was demonstrated on MCF-7 and MDA-MB-231 cells with IC_50_ values of 50 μg∙mL^−1^ and 44 μg∙mL^−1^, respectively [61].

The results of our research of 20 lichen species showed that the cell line HL-60/MX2 are sensitive to the effects of tested extracts to varying degrees. Generally, the anticancer activity of studied extracts was rather poor, because the lowest values of IC_50_ [µg∙mL^−1^]—99.4 (for HP1), 122.6 (CDIG), 136.5 (HP), 142.6 (CS), and 180.2 (CM1). The rest of the samples show weaker activity, the value of IC_50_ are higher than 200.

### 2.6. Partial Least Squares

**Figure 3:**
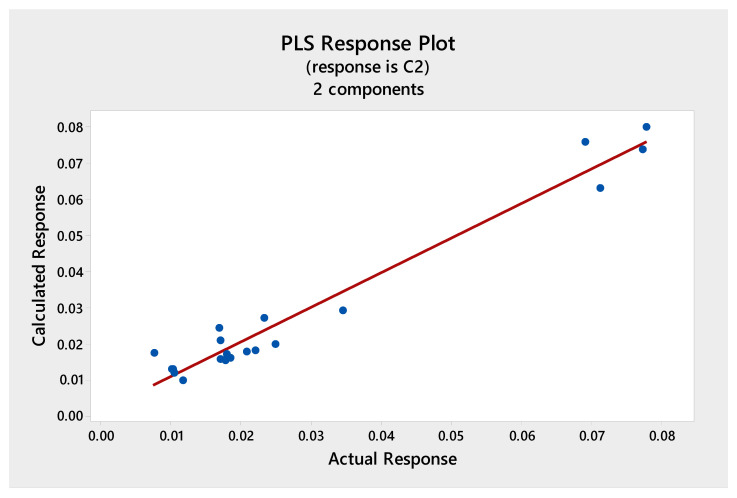
The Partial Least Squares (PLS) correlation graph for predicted and measured values of the antioxidant activity (DPPH method). Regression fit, Fitted = 0.0012 + 0.9596 Actual, R-Sq = 96.0%.

**Figure 4:**
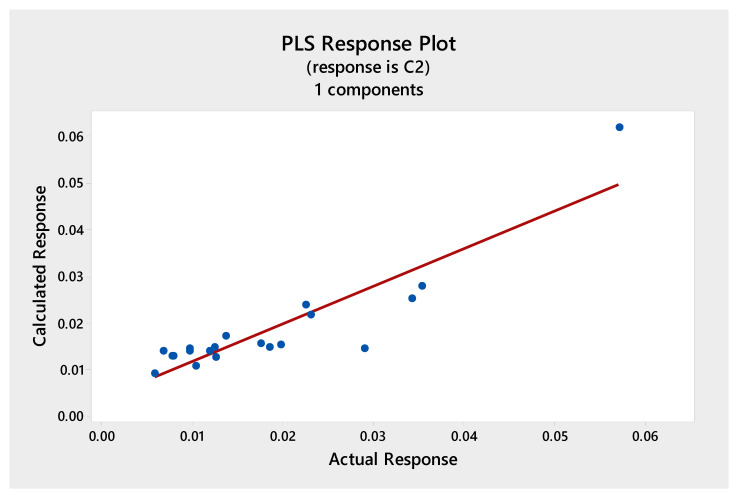
The PLS correlation graph for predicted and measured values of the antioxidant activity (FRAP method). Regression fit, Fitted = 0.0034 + 0.8095 Actual, R-Sq = 81.0%.

**Figure 5:**
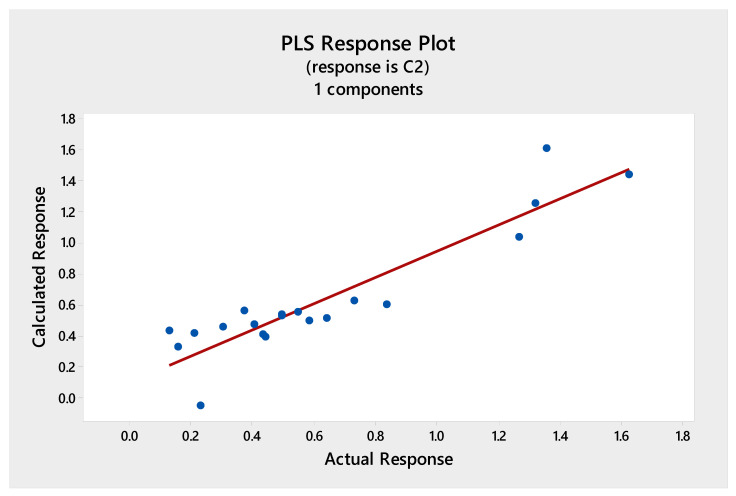
The PLS correlation graph for predicted and measured values of the phenolics content (F-C method). Regression fit, Fitted = 0.0948 + 0.8479 Actual, R-Sq = 84.8%.

**Figure 6:**
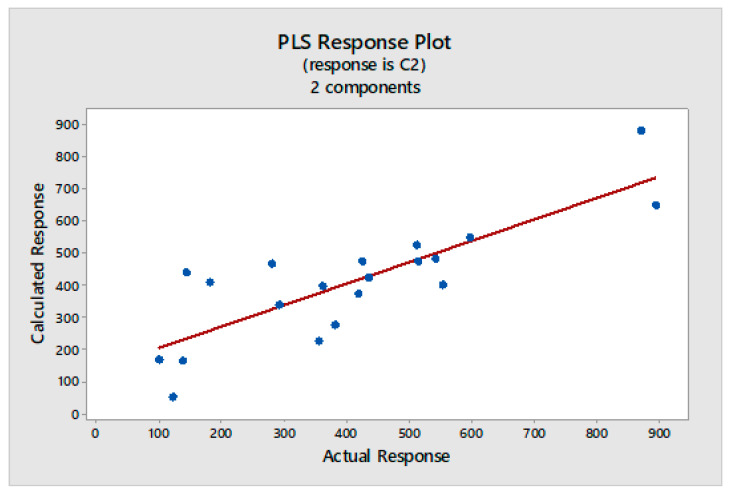
The PLS correlation graph for predicted and measured values of the cytotoxic effect. Regression fit, Fitted = 133.8 + 0.6700 Actual, R-Sq = 67.0%.

Multivariate calibration models were constructed with the data matrix consisting of the 20 fingerprint chromatograms and the response vectors presenting the results from the total phenolics, antioxidant, or cytotoxic activities. The independent matrix comprises the representation of the HPLC data and the dependent matrix comprises the measured total phenolics, antioxidant, or cytotoxic activities. The relationships were presented in Figure 3, Figure 4, Figure 5 and Figure 6, as scatterplots of real data versus data computed with the trained PLS model. 

Figure 3 shows the calibration graph for the ability to scavenge free radicals obtained using DPPH test. The high value of determination coefficient (*r*^2^ = 0.96) between the predicted (calculated) and actual (obtained based on the chromatographic data) antioxidant activity confirms good prediction of used calibration model. 

The prediction of antioxidant properties based on lichen chromatograms was also performed for the FRAP method and the obtained calibration model was presented in Figure 4. In this case the determination coefficient of the calculated (predicted) and actual (determined based on chromatographic data) responses was slightly lower, *r*^2^ = 0.81. However, this PLS model also could be applied to predict the antioxidant activity of studied lichens based on their phytochemical profiles.

The PLS model, which predicts the phenolic content in selected lichen extracts, was also built for the F-C test and the graph was presented in Figure 5. As reported in the literature [62,63,64,65,66] the polyphenol content in plant extracts was correlated with their antioxidant activity. As expected, the determination coefficient of the obtained calibration curve was numerically similar to DPPH and FRAP tests and was equal *r*^2^ = 0.85. The obtained result confirms the usefulness of this method for the predicting phenolic compounds content in lichen extracts based on their phytochemical profiles.

In this study the prediction of the cytotoxic activity of studied lichen extracts based on their chromatographic profiles was also confirmed and the graph was presented in Figure 6. The value of determination coefficient for this model was *r*^2^ = 0.67. This weaker correlation confirms the limited usefulness of created PLS model for prediction of the anticancer activity of studied lichen extracts based on the chromatographic data.

In all cases, one or two PLS components were enough to build a well-working, non-overfitted model. This proves that the information about the investigated properties was located inside the fingerprints and its structure was not so complex, as there was no need to incorporate further subsequent components into the model. The dependence between the real and fitted property was highly correlated for DPPH, FRAP, and F-C methods (Figure 3, Figure 4 and Figure 5) and a weaker correlation was obtained for cytotoxic activity test (Figure 6).

## 3. Materials and Methods

### 3.1. Plant Material

Twenty lichen species (Table 1) were collected from natural sites in two voivodships—Lubelskie and Małopolskie (Poland). The identification of species was performed by Dr. Hanna Wójciak (Department of Botany and Mycology, Maria Curie-Skłodowska University in Lublin) via spot tests reactions, macroscopic, and microscopic examinations. The names, symbols, and time of harvest of plant material were presented in Table 1. All samples were from Lublin province, and the following lichens (presented as symbols) originate from Sobibór Landscape Park—HP, EP, CM, CF; from Kozłowieckie Forests—HP1; from Kodeń—PF, CE, CI1; from Leszkowice—CI, CM1, CFIM, CS, CC, CD; from Janowskie Forests—CRAN, CSYL, CCRI from Nowa Biała—CDIG; from Dębowiec—CO; from Gronków—XP. 

Among the examined lichens there are species under partial protection, in accordance with the Regulation of the Minister of the Environment of 9 October 2014 on the protection of species of mushrooms, i.e., *Cladina arbuscula* (Wallr.) Flotow, *Cladina rangiferina* (L.) Weber ex Wigg., *Cetraria islandica* (L.) Ah. and *Cetraria ericetorum* Opiz. The permission of the Regional Director for Environmental Protection in Lublin was obtained for the collection of these lichens.

Three species i.e., *Cetraria islandica* (L.) Ach., *Cladina mitis* and *Hypogymnia physodes* were collected from two, and *Cladonia furcata* (Huds.) Schrader from three different positions. In total, 26 samples were obtained, of which extracts were made.

Organic and mineral impurities were manually removed from the raw plant material, and air-dried at room temperature for a week. Next, they were grouped according to species affiliation.

### 3.2. Preparation of Extracts 

Dried and purified raw materials were ground in a laboratory mill (IKA®-WERKE, Staufen, Germany) and stored in refrigerator (tightly closed) from one to eight months (depending on the harvest time). Then 10 g portion of each raw material was placed in a cellulose case (in the case of *Xanthoria parietina* (L.) Th. Fr. and *Cladonia subulata* (L.) Weber ex Wigg. the smaller amounts of thallus were collected—about 8 g and 4 g, respectively) (Table 4). The extraction was carried out in a Soxhlet apparatus (200mL, SIMAX, KAVALIERGLASS, Prague, Czech Republic) in two stages using dichloromethane (DCM, Polish Reagents, Gliwice, Poland) and next methanol (MeOH, Polish Reagents, Gliwice, Poland) as solvents. Each solvent (DCM, MeOH) was used in an amount of 300 mL. Both DCM and MeOH extraction process lasted 8 h each. In this work the methanolic extracts were analyzed.

The methanolic extracts were evaporated to dryness in a vacuum evaporator (IKA®-WERKE, Staufen, Germany), and the dry residues (Table 4) were transferred quantitatively into volumetric flasks with a capacity of 50 mL and made up to the mark with ethanol. The extracts were poured into amber glass bottles, sealed, and stored in a refrigerator at 2–8 °C.

### 3.3. High Performance Liquid Chromatograpy (HPLC) Conditions and the Description of Standard (Usnic Acid)

HPLC analysis gradient elution was performed using HP 1100 Agilent chromatograph (Agilent, Santa Clara, CA, USA) with ChemStation for LC 3D Systems Rev. B.04.03, equipped with: a four-component gradient pump (1100 Series Quaternary Pump, G1311A); autosampler (1100 Series Autosampler, G1313A); UV-VIS detector with diode strip (1100 Series Diode Array Detector, G1315A); column thermostat (1100 Series Thermostatted Column Compartment, G1316A); degasser (1100 Series Vacuum Degasser, G1322A). Phenomenex KINETEX^®^ EVO C-18 chromatographic column (250 mm, 4.6 mm, pore size 100 Å, grain diameter 5 µm, protected silanol group, Torrence, CA, USA) was used as stationary phase. The mobile phase consisted of methanol (pure for HPLC, ROMIL, Cambridge, UK), water (distilled and purified water was obtained using the HYDROLAB 5 water treatment system, model: HLP 5 UV) and phosphoric acid (pure per analysis, 85 % (*m*/*m*), J. T. Baker, Center Valley, PA, USA). The gradient of methanol-solvent B and water acidified with phosphoric acid (V) (pH 2.3)-solvent A was applied according to program: 0 min—50% A + 50% B, 30 min—1% A + 99% B, 50 min—1% A + 99% B. The time of analysis was 50 min, the temperature of column thermostat was 22 °C, the mobile phase flow was 1 mL·min^−1^ and the analytical wavelength was λ = 302 nm.

Each of sample was filtered using Econofilter 25 mm syringe filters with PTFE membrane with 0.45 μm pore size (Agilent, Santa Clara, CA, USA) and located in an autosampler. The injection volume was 20 µL.

Usnic acid ((+)-usnic acid 98% (Sigma-Aldrich, St. Louis, MO, USA) was used as standard in qualitative and quantitative analysis using chromatographic techniques. In order to identify usnic acid during the HPLC analysis of the tested extracts, the marker of usnic acid was used (it was used for qualitative and quantitative analysis of products from Icelandic lichen *Cetraria islandica*. The retention time of (+)-usnic acid in the indicated analysis conditions was 23.978 min.

### 3.4. Antioxidant Activity

The in-vitro antioxidant activity of selected lichens was determined by spectrophotometric technique (Spectrfotometer GENESYS ™ 20, THERMO SPECTRONIC, Rochester, NY 14625, USA) using the following reagents: 2,2-diphenyl-1-picrylhydrazyl (DPPH, Sigma-Aldrich, St. Louis, MO, USA), 2,4,6-tris(2-pyridyl)-1,3,5-triazine (TPTZ, Sigma-Aldrich, St. Louis, MO, USA). The total phenolic content was determined using Folin-Ciocalteu reagent (F-C, Sigma-Aldrich, St. Louis, USA) and trolox ((±)-6-hydroxy-2,5,7,8-tetramethylchroman-2-carboxylic acid (Sigma-Aldrich, St. Louis, MO, USA) as standard was used.

#### 3.4.1. DPPH Test

Preparation of the test solutions: Ethanolic DPPH solution was prepared by dissolving 19.71 mg DPPH in 100 mL of ethanol. The solution was diluted so that the absorbance was about 0.9 at the wavelength λ = 517 nm. The reagent was stored in a dark and cool place for no more than 3 days. Control test was prepared by mixing 1.5 mL of DPPH solution and 0.02 mL of ethanol and the test sample was prepared by mixing 1.5 mL of DPPH solution and 0.02 mL of a given extract. The concentration of trolox solution was 0.25 g∙mL^−1^ and the dilutions were prepared (mg∙mL^−1^): 0.125, 0.05, 0.03125, 0.025, 0.0125, 0.00625, 0.003125. The following values of absorbance (AU) and inhibition (%) were obtained for the above concentrations, respectively: 0.410 and 56.44; 0.610 and 34.27; 0.702 and 24.35; 0.787 and 15.19; 0.883 and 4.89; 0.925 and 0.36.

Spectrophotometric determination of antioxidant activity using the DPPH radical is carried out according to the modified Brand-Williams method [67]. The activity of the tested antioxidant is given as a percentage of inhibition (degree of scavenging of the radical) and is calculated from formula:

(1) $$\%\;inhibition\;=\;100\times \frac{\left(A_{0}-A\right)}{A_{0}}$$

where: *A*_0_ is the absorbance of DPPH radical solution, *A* is the average of the absorbance value of the test solution containing antioxidant.

All measurements were made at a wavelength λ = 517 nm using quartz cuvettes in a Genesis 20 spectrophotometer against ethanol as a blank. The absorbance of control *A*_0_ was measured for three samples, respectively, and the average was calculated. The tested samples were placed in a dark place. After 30 min, absorbance was measured. Three tests were performed for each extract. Antioxidant activity was calculated and expressed as % inhibition.

#### 3.4.2. FRAP (Ferric Reducing Antioxidant Power)

The assay procedure involves determining the ability to reduce iron ions Fe(III) to Fe(II), which with TPTZ form a complex with a navy-blue color with a maximum absorbance at 593 nm. Quantitative determinations of the tested samples are carried out using the standard curve method based on the absorbance measurements of trolox or gallic acid solutions [68]. The following test solutions were prepared: (1) 300 mM acetate buffer solution at pH 3.6; (2) 40 mM hydrochloric acid solution; (3) 10 mM TPTZ solution; (4) 20 mM iron (III) chloride solution FRAP reagent was prepared by mixing 41.67 mL 20 mM iron (III) chloride and 41.67 mL 10 mM TPTZ, then the mixture was transferred quantitatively into a 500 mL flask and made up to volume with acetate buffer. Control sample was prepared by mixing 50 μL distilled water and 1450 μL FRAP reagent. Test sample was prepared by mixing 50 μL of a given extract and 1450 μL of FRAP reagent. Three samples were made. The following dilutions were prepared based on trolox stock solution (c = 0.25 mg∙mL^−1^): 0.03125, 0.025, 0.0125, 0.006, 0.003, 0.0016, 0.0008. Measurements were made at a wavelength λ = 593 nm using quartz cuvettes. The control (50 μL distilled water + 1450 μL FRAP reagent) was prepared for 8 min after preparation, then its absorbance was measured. The test samples were conducted in a same manner—after preparation, the solutions were allowed to stand for 8 min, after which the absorbance was measured. 

### 3.5. Total Phenolic Content with Folin-Ciocalteu Reagent

The Folin-Ciocalteu (F-C) method [69,70] allows to determine the total phenolic content using the F-C reagent. The basis of the determination is the reversible reduction reaction by molybdenum (VI) phenols to molybdenum (V) contained in the F-C reagent in an alkaline medium. As a result of the reaction, a blue compound is formed that shows a maximum absorbance at λ = 745–750 nm. 

Preparation of test solutions: control sample was prepared by mixing 1 mL of distilled water 100 µL F-C reagent in tube. After 5 min, 1 mL sodium carbonate (75 g·L^−1^) and 400 µL distilled water were added and located in the dark for 2 h. Test sample was prepared by mixing 50 μL of a given extract, 950 μL of distilled water and 100 μL of F-C reagent in test tube. After 5 min, 1 mL sodium carbonate (75 g·L^−1^) and 400 µL distilled water were added and placed in the dark for 2 h. The concentration of gallic acid standard solutions was equal to 1 mg∙mL^−1^. The stock solution was diluted, and the following concentrations were obtained (mg∙mL^−1^): 0.5, 0.25, 0.125, 0.01. All measurements were performed at a wavelength λ = 760 nm using quartz cuvettes. The absorbance of gallic acid standard solutions against the blank was measured. On this basis, a calibration curve of absorbance versus concentration was plotted. The absorbance measurements were then carried out for all tested extracts against the blank. The content of polyphenols in individual extracts (mg∙mL^−1^) was calculated from the plotted gallic acid calibration curve.

### 3.6. Assessment of Cytotoxicity by Trypan Blue Staining

A method of directly measuring cell viability in culture, i.e. determining the number of live or dead cells using appropriate dyes, can be used for these studies. Colored substances either penetrate inside the cell or remain on its surface depending on the state of the cell membrane. Trypan blue (BT, 0.4% Trypan Blue solution, Bio-Rad Laboratories Ltd., Watford, UK) was used in this work, which only stains dead cells, late-apoptotic, or necrotic, which was associated with a change in the integrity of the cell membrane; it does not penetrate into the cytoplasm of living cells. Cells with a dark blue cell membrane are counted using an automatic counter or hemocytometer in a light microscope.

#### 3.6.1. Cell Line

The HL-60/MX2 human cancer cell line of acute promyelocytic leukemia (CRL-2257) was used to conduct scheduled studies and assess cytotoxicity (American Type Culture Collection, ATCC 10801 University Boulevard Manassas, VA 20110 USA).

Cultures were grown under standard conditions in RPMI 1640 (PAA Laboratories, Linz, Austria) medium supplemented with 10% addition of fetal bovine serum (FBS, PAA Laboratories, Linz, Austria) and with antibiotics penicillin (PAA Laboratories, Linz, Austria) at 100 U·mL^−1^, streptomycin (PAA Laboratories, Linz, Austria) at 100 µg·mL^−1^ and amphotericin (PAA Laboratories, Linz, Austria) at 2.5 µg·mL^−1^. The cell suspension density was measured using the Bio-Rad TC 10™ Automated Cell Counter.

Cells of the tested lines were passaged and seeded in 12-well culture plates (Sarstedt, Nümbrecht Germany) and incubated for 24 h, and then exposed to increasing extract concentrations (µL), i.e., 1, 5, 10, 25, and 50 doses of each of the original extracts were supplemented with 1 mL buffered saline (PBS, Sigma-Aldrich (St. Louis, MO, USA). Then another incubation was carried out for 24 h. Cells were centrifuged (800 rpm, 5 min), washed with PBS and centrifuged again, and then stained with 0.4 % trypan blue solution (Bio-Rad Laboratories Ltd., Watford, UK) and counted using a TC 10™ Automated Cell Counter (Bio-Rad Laboratories Ltd., Watford, UK) following the manufacturer’s procedure. Dead cells stain blue because their cell membrane was damaged, and the dye penetrates inside the cell. Each test was performed in duplicate.

The control was unstimulated cells. To determine the IC_50_ value, a graph was made of the dependence of cell viability (expressed as a percentage relative to the control for which 100% was assumed) on the concentration of the tested extract. The value of the first sample was temporarily removed from the data, and the values for the remaining samples are used to calculate the regression equation. This new equation was then used to estimate (predict) the variable value for the omitted sample. The procedure was repeated, omitting one more sample at a time.

### 3.7. Chemometric Analysis

#### 3.7.1. Data Analysis and Pretreatment Chromatographic Data

The chromatographic data processing was proceeded in several stages. At the beginning, 20 chromatograms of the studied lichens were exported as the text files (ASCII) with the extension .asc. Then the received data was copied to Excel using a text editor. The analysis was carried out in the 200–400 nm wavelength range, while λ = 302 nm was selected for chemometric treatment. The next step was to calculate the arithmetic mean of the data in each line (three chromatograms were obtained for each sample of extract) and the matrix consisting of (20 columns (chromatograms) and 7501 lines) (time points from 0 to 50 min in 7501 steps) of the retention times and absorbance appropriate was created.

Then, the obtained data was standardized so that the sum of all components in the PCA analysis was 100% and The MiniTab18 program (Minitab, State College, PA, USA) was used for this purpose. The obtained data matrix from excel was copied and standardized (autoscaled). Next, normalized data was copied to excel and saved as .csv (comma-separated value). In the next stage, the data was subjected to the alignment process using the SpecAlign program (developed by Dr. Jason Wong, University of Oxford, Oxford, UK). A baseline was then generated with a window width (parameter of the built-in algorithm) of 20 that and it was subtracted. Then, the data smoothing and denoising were performed using the Savitzky-Golay algorithm with the window size (also a parameter of this filter) equal to −8. Next, the alignment of chromatograms was performed using the recursive alignment by fast Fourier transform algorithm [71]. The synchronization was performed with max Shift equal to 20, and the obtained data were exported as csv to Excel.

#### 3.7.2. Principal Component Analysis (PCA)

Principal component analysis as a widely available unsupervised pattern recognition technology was applied to the preliminary evaluation of the chemical similarity between the fingerprint HPLC chromatograms. PCA is the most commonly used tool for exploratory data analysis and it was conducted to efficiently reduce the dimensions of the original (chromatographic) data set to a smaller number of principal components without much information loss [72]. PCA calculations was performed using the Statistica software (version 13.3).

#### 3.7.3. Correlation Between Biological Activity and Chromatographic Fingerprint by PLS 

Partial Least Squares is linear regression algorithm commonly used to model the best combination of descriptor variables X (data set) to predict or evaluate the property variable Y (response set) [73]. In our paper, PLS was performed to combine data from the biological activities (antioxidant and anticancer) and chemical fingerprints of lichen methanolic extracts. These calculations enabled us to establish the fingerprint activity relationship using Minitab software (version 18), for the future study of bioactive compounds responsible for antioxidant activity of lichens extracts. To select the optimum number of factors to be used in the PLS calibration model a cross-validation with "leave-one-out" procedure was used. Then the difference between the measured and the predicted value of the variable is calculated for each sample. The sum of squares of these differences is called the predicted residual error sum of squares or PRESS for short. When the value of the PRESS statistic is closer to zero, it shows the better forecasting ability of the model.

The established model was used to predict the antioxidant and cytotoxic properties of selected lichen methanolic extracts from their chromatographic profiles. The results were compared with the results obtained experimentally.

## 4. Conclusions

In this study RP-HPLC analysis of selected lichens methanolic extracts were performed to obtain their fingerprint profiles. The chemical similarity between the same lichen species collected from various natural sites (*Cetraria islandica*, *Cladina mitis*, *Hypogymnia physodes*) was confirmed using Principal Components Analysis. 

Additionally, the biological activities (antioxidant and anticancer) were determined and evaluated. The methanolic extracts of the following lichens: *Hypogymnia physodes*, *Cladonia digitata* (L.) Hoffm., *Pseudevernia furfuracea* (L.) Zopf. show highest ability to scavenge free radicals (DPPH and FRAP tests), while *Cladonia furcata* (Huds.) Schrader (DPPH) and *Cladina arbuscula (Wallr.) Flotow* (FRAP test) have a lowest antioxidant activity.

As expected, the highest content of phenolics was observed in the same lichen species (*Pseudevernia furfuracea* (L.) Zopf., *Cladonia digitata* (L.) Hoffm., *Hypogymnia physodes*. The strongest cytotoxic activity (the lowest value of IC_50_) show the following lichen extracts: *Hypogymnia physodes*, *Cladonia digitata* (L.) Hoffm., *Cladonia subulata* (L.) Weber ex Wigg. and *Cladina mitis.*

A simple PLS calibration model was applied to predict the biological activities of studied samples based on their chromatographic fingerprint profiles. In four built models, one or two PLS components were enough to build a well-working, non-overfitted model. This confirmed that the information about the biological properties was located inside the fingerprints and its structure is was not so complex, as there was no need to incorporate further subsequent components into the model. The obtained relationships between the actual (determined based on chromatographic data) and fitted (predicted) properties were highly correlated in the case of antioxidant activity tests and weaker for the cytotoxic test. 

This work confirms that the combination of fingerprint chromatography, qualitative analysis, and multidimensional chemometric techniques (PCA and PLS) can be used as reliable and economic quality control tool for lichens in commercial or herbal products as well as any plant material. After the preliminary qualitative research and fingerprint analysis, the next stage of research in search of new biologically active substances will be the LC-MS analysis.

## Figures and Tables

**Table 1:** The names, symbols, and time of harvest of selected lichens.

No.	Name	Symbol	Time of Harvest
1.	*Hypogymnia physodes* (L.) Nyl.	HP	X. 2016
2.		HP1	IX. 2016
3.	*Evernia prunastri* (L.) Ach.	EP	X. 2016
4.	*Pseudevernia furfuracea* (L.) Zopf	PF	XI. 2016
5.	* Cetaria ericetorum Opiz	CE	XI. 2016
6.	* Cetraria islandica (L.) Ach.	CI	XI. 2016
7.		CI1	XI. 2016
8.	* Cladina rangiferina (L.) Weber ex Wigg. *synonym Cladonia rangiferina* (L.) Nyl.	CRAN	V. 2017
9.	* Cladina arbuscula (Wallr.) Flotow *synonym Cladonia sylvatica auct*	CSYL	V. 2017
10.	*Cladina mitis Sandst. Synonym C. mitis (Sandst.) Hustich* (1951), *C. arbuscula subsp. mitis* (Sandst.) Ruoss (1987)	CM	X. 2016
11.		CM1	XI. 2016
12.	*Cladonia digitata* (L.) Hoffm.	CDIG	IV 2017
13.	*Cladonia fimbriata* (L.) Fr. *synonym C. major* (Hagen) Sandst., *C. minor* (Hagen) Vainio	CFIM	XI. 2016
14.	*Cladonia ochrochlora* Flk.	CO	IV. 2017
15.	*Cladonia subulata (L.) Weber ex Wigg.* *synonym C. cornutoradiata (Coem.) Zopf.*	CS	XI. 2016
16.	*Cladonia cornuta* (L.) Hoffm.	CC	XI. 2016
17.	*Cladonia furcata* (Huds.) Schrader	CF	X. 2016
18.	*Cladonia crispata* (Delise) Vainio	CCRI	V. 2017
19.	*Cladonia phyllophora* Hoffm. *synonym C. degenerans* (Flk.)	CD	XI. 2016
20.	*Xanthoria parietina* (L.) Th. Fr.	XP	IV. 2017

* species under partial protection.

**Table 2:** Inhibition %, the concentrations expressed as trolox (concentration of trolox equivalent—C_TE_) equivalents for 2,2-diphenyl-1-picrylhydrazyl (DPPH) and ferric reducing antioxidant power (FRAP) methods, and expressed as gallic acid (concentration of gallic acid equivalent—C_GAE_) equivalent for Folin-Ciocalteu (F-C) test. Symbols of lichen samples according to Table 1.

	DPPH		FRAP	F-C Test
Sample	Inhibition [%]	C_TE_ [mg∙mL^−1^] (trolox)	C_TE_ [mg∙mL^−1^] (trolox)	C_GAE_ [mg∙mL^−1^] (gallic acid)
**EP**	19.08	0.0185 ± 0.002	0.0119 ± 0.001	0.496 ± 0.061
**PF**	77.08	0.0690 ± 0.000	0.0225 ± 0.002	1.623 ± 0.067
**CE**	26.01	0.0221 ± 0.001	0.0127 ± 0.001	0.230 ± 0.018
CS *	24.57	0.0105 ± 0.001	0.00975 ± 0.001	0.211 ± 0.012
**CC**	17.10	0.0168 ± 0.003	0.0138 ± 0.001	0.3720 ± 0.093
**CFIM**	21.57	0.0180 ± 0.002	0.0176 ± 0.001	0.405 ± 0.008
**CSYL**	18.84	0.0177 ± 0.003	0.0059 ± 0.001	0.444 ± 0.060
**CDIG**	86.72	0.0771 ± 0.000	0.0571 ± 0.001	1.355 ± 0.093
**CCRI**	22.27	0.0207 ± 0.001	0.0104 ± 0.001	0.586 ± 0.021
**CRAN**	17.99	0.0170 ± 0.003	0.0125 ± 0.001	0.547 ± 0.013
XP *	24.85	0.0233 ± 0.002	0.0231 ± 0.001	0.4338 ± 0.046
**CO**	38.04	0.0344 ± 0.001	0.0198 ± 0.002	0.730 ± 0.091
**CI**	29.16	0.0249 ± 0.000	0.0185 ± 0.001	0.835 ± 0.143
**CI1**	12.86	0.0102 ± 0.001	0.0290 ± 0.000	0.640 ± 0.022
**CM**	12.03	0.0117 ± 0.002	0.0078 ± 0.001	0.159 ± 0.018
**CM1**	18.02	0.0170 ± 0.002	0.0068 ± 0.001	0.131 ± 0.022
**CD**	12.90	0.0103 ± 0.002	0.0080 ± 0.001	0.305 ± 0.055
**CF**	10.02	0.0076 ± 0.001	0.0098 ± 0.001	0.494 ± 0.047
**HP**	80.31	0.0711 ± 0.001	0.0342 ± 0.001	1.264 ± 0.019
**HP1**	87.29	0.0778 ± 0.000	0.0353 ± 0.001	1.317 ± 0.013

* The values of % CS and % XP inhibition were multiplied by 2.5 and 1.25 (respectively) due to 2.5 and 1.25 times (respectively) lower mass of the raw material used for extraction in relation to the rest of the species.

**Table 3:** IC_50_ values for studied lichen samples—trypan blue method. Symbols according to Table 1.

Sample	IC_50_ [µg∙mL^−1^]	Sample	IC_50_ [µg∙mL^−1^]
**EP**	355.3	**XP**	553.3
**PF**	381.5	**CO**	511.3
**CE**	433.6	**CI**	514.0
**CS**	142.6	**CI1**	541.8
**CC**	894.3	**CM**	418.4
**CFIM**	870.2	**CM1**	180.2
**CSYL**	361.6	**CD**	596.6
**CDIG**	122.6	**CF**	279.1
**CCRI**	425.4	**HP**	136.5
**CRAN**	292.5	**HP1**	99.4

**Table 4:** Sample weight and dry residue masses of studied lichen samples after dichloromethane and methanolic extractions. Symbols of lichens according to Table 1.

Symbol	Sample Weight [g]	Dry Residue [g]
EP	9.9998	0.8276
PF	10.0421	2.0540
CE	9.9847	0.6504
CS	3.9996	0.1935
CC	10.0835	1.1382
CFIM	10.0274	0.9896
CG	10.0026	0.0668
CSYL	10.0300	1.0000
CDIG	10.0300	1.4500
CRAN	10.1800	0.7200
XP	7.9800	1.3300
CO	10.0400	2.8900
CI	10.2897	1.1090
CI1	10.0303	1.1533
CM	10.0026	0.4339
CM1	10.0151	0.3604
CD	10.0742	0.7670
CF	10.0035	0.6663
HP	10.0091	0.7498
HP1	10.0569	0.5860

## Supplementary Materials

Figure S1: The chromatogram of *Cladina mitis* (CM) methanolic extract, UA–usnic acid.; Figure S2: The chromatogram of *Cladina mitis* (CM1) methanolic extract, UA–usnic acid.; Figure S3: The chromatogram of *Cladina arbuscula* (CSYL) methanolic extract, UA–usnic acid.; Figure S4: The chromatogram of *Evernia prunastri* (EP) methanolic extract, UA–usnic acid.; Figure S5: The chromatogram of *Hypogymnia physodes* (HP) methanolic extract, UA–usnic acid.; Figure S6: The chromatogram of *Hypogymnia physodes* (HP1) methanolic extract, UA–usnic acid.; Figure S7: The chromatogram of *Xanthoria parietina* (XP) methanolic extract, UA–usnic acid.
