# Supplementary material for: HPLC Fingerprint Analysis with the Antioxidant and Cytotoxic Activities of Selected Lichens Combined with the Chemometric Calculations
Source: Molecules. 2020 Sep 19;25(18):4301. doi: 10.3390/molecules25184301 (PMC7571045; doi:10.3390/molecules25184301)
Supplement: Supplementary file 1 [file molecules-25-04301-s001.pdf]

Supplementary Material

HPLC Fingerprint Analysis with the Antioxidant and Cytotoxic Activities of Selected Lichens Combined with the Chemometric Calculations

Anna Hawrył [1], Mirosław Hawrył [1,*], Agnieszka Hajnos-Stolarz [2], Jagoda Abramek [3], Anna Bogucka-Kocka [3] and Łukasz Komsta [4]

[1] Department of Inorganic Chemistry, Faculty of Pharmacy, Medical University of Lublin, Chodźki 4a St. 20-093 Lublin, Poland; anna.hawryl@umlub.pl (A.H.)
[2] Herbapol-Lublin S.A., Diamentowa 25 Street, 20-471 Lublin, Poland; ahajnos@gmail.com (A.H.-S.)
[3] Department of Biology and Genetics, Medical University, Chodźki 4A Street, 20-093 Lublin, Poland; anna.bogucka-kocka@umlub.pl (A. B.-K.), jagoda.abramek@umlub.pl (J.A.)
[4] Chair and Department of Drug Chemistry, Medical University, Jaczewskiego 4 Street, 20-090 Lublin, Poland; lukasz.komsta@umlub.pl (L.K.)
* Correspondence: mirek.hawryl@umlub.pl (M.H.)

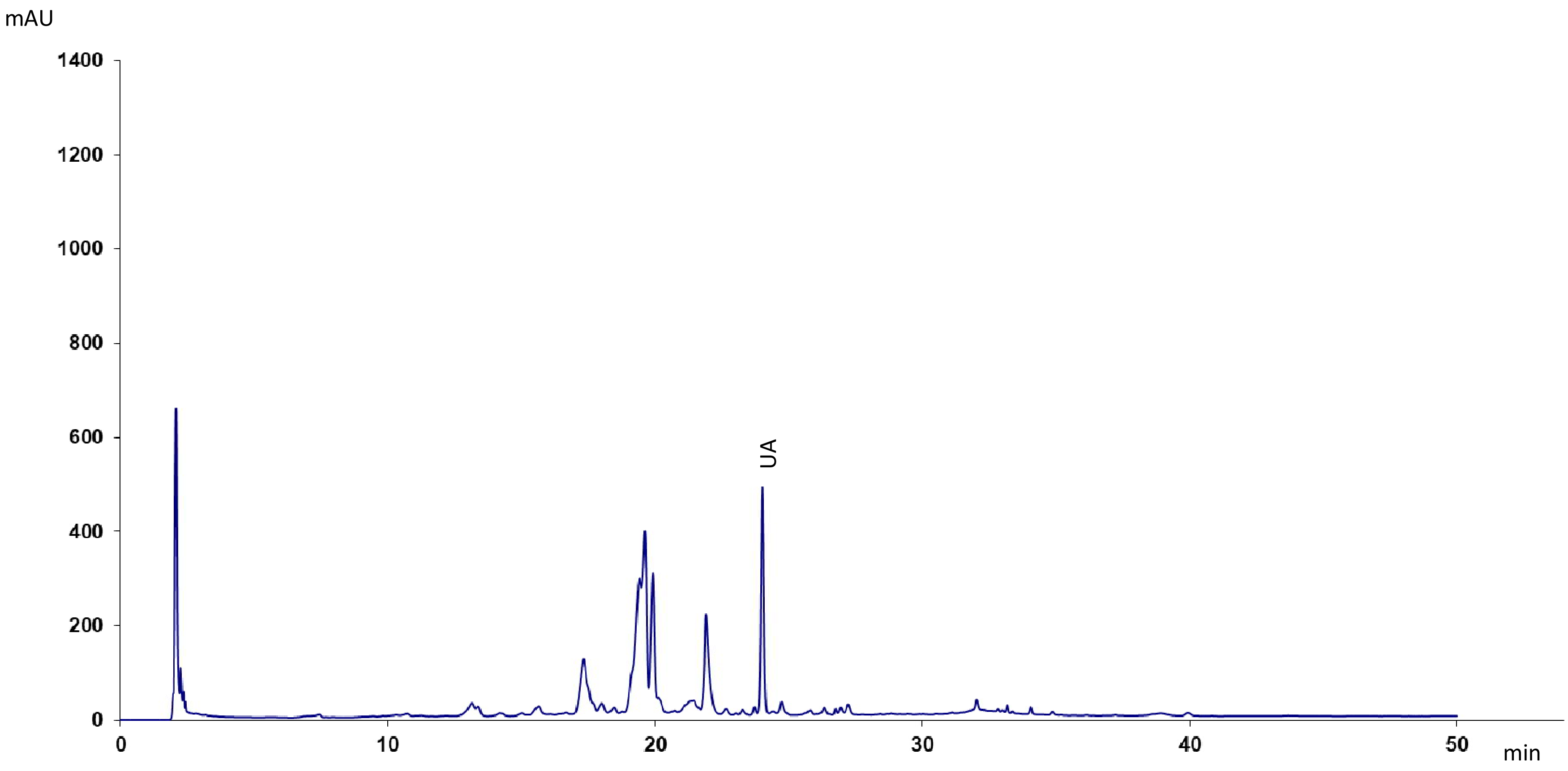

Figure S1. The chromatogram of *Cladina mitis* (CM) methanolic extract, UA – usnic acid.

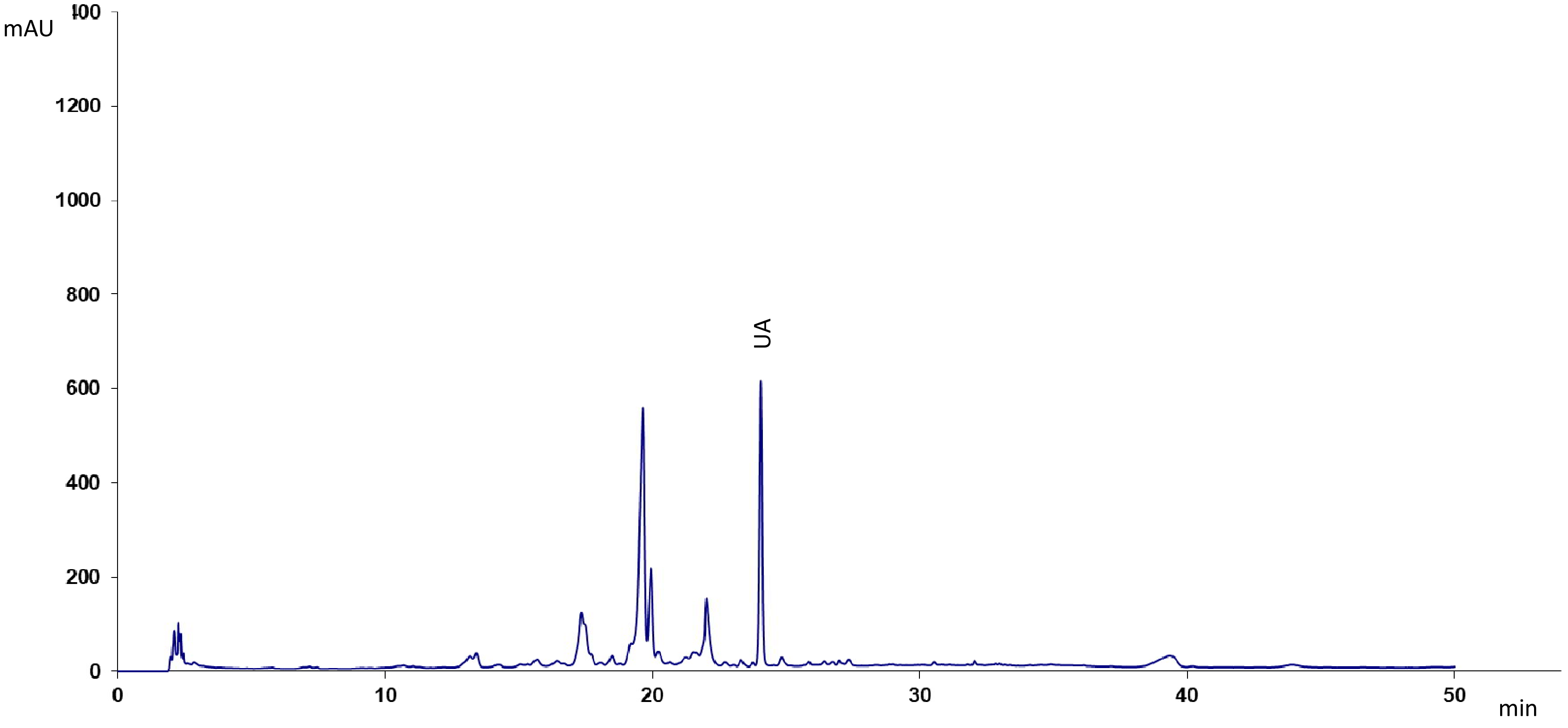

Figure S2. The chromatogram of *Cladina mitis* (CM1) methanolic extract, UA – usnic acid.

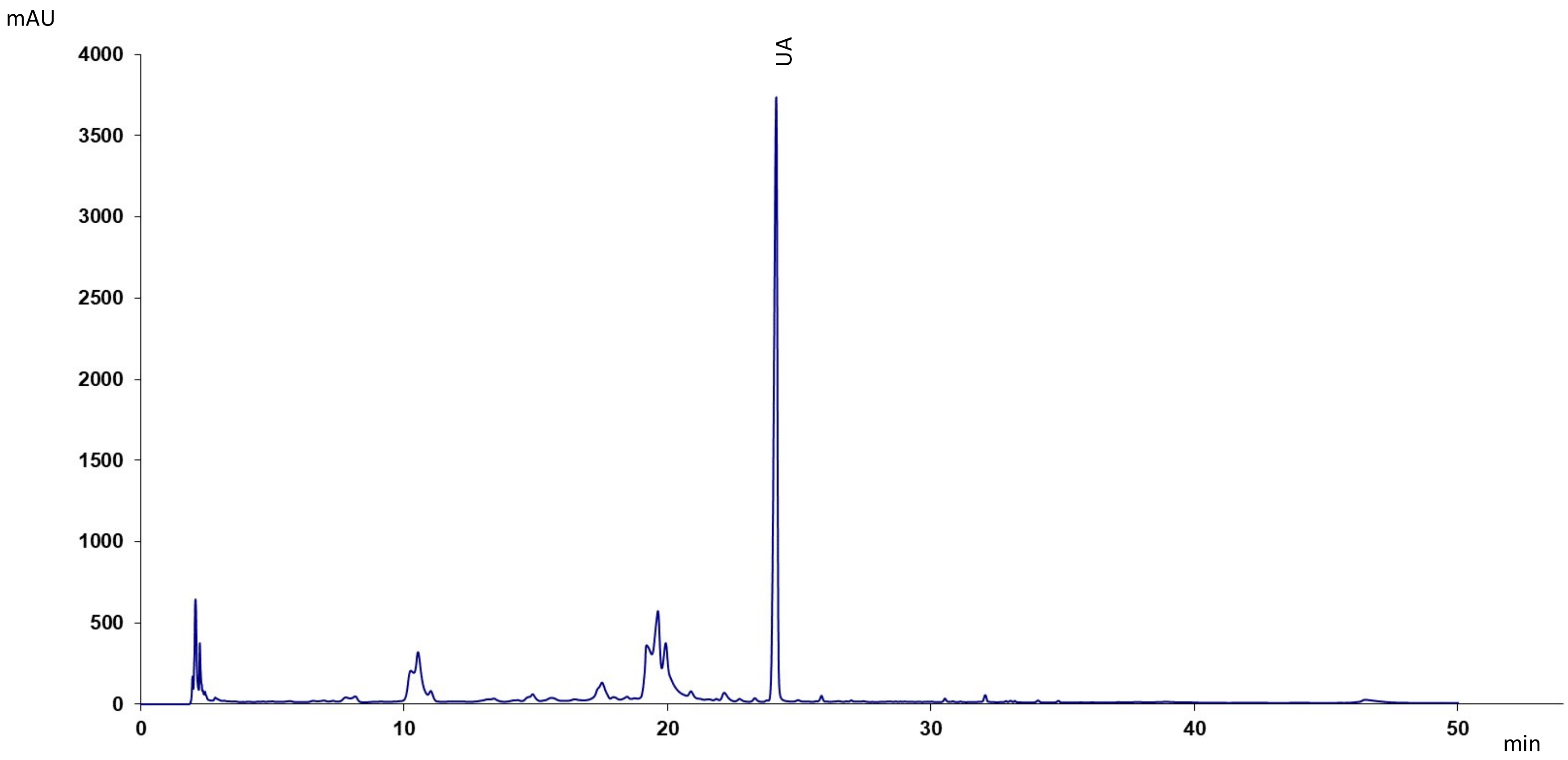

Figure S3. The chromatogram of *Cladina arbuscula* (CSYL) methanolic extract, UA – usnic acid.

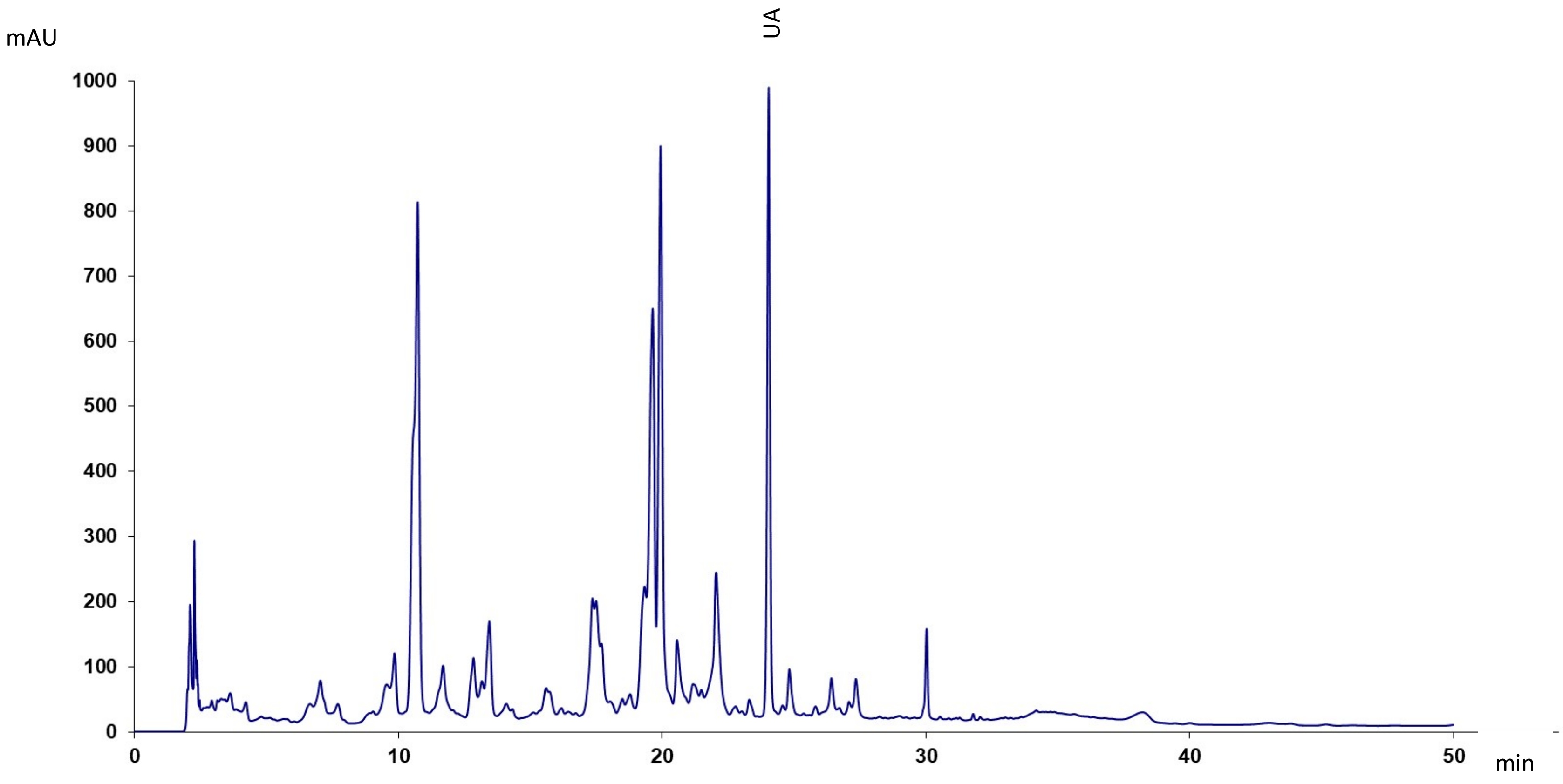

Figure S4. The chromatogram of *Evernia prunastri* (EP) methanolic extract, UA – usnic acid.

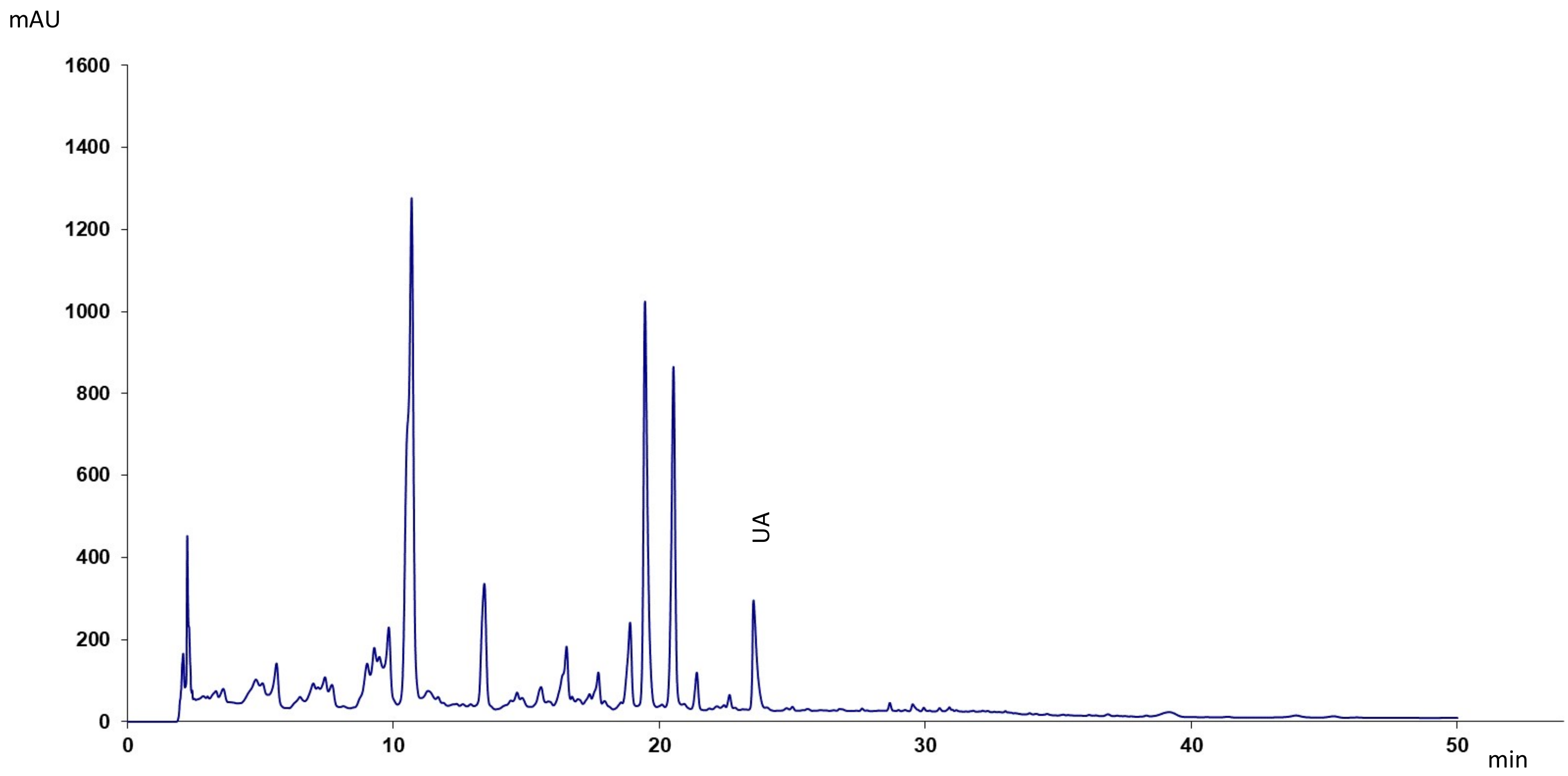

Figure S5. The chromatogram of *Hypogymnia physodes* (HP) methanolic extract, UA – usnic acid.

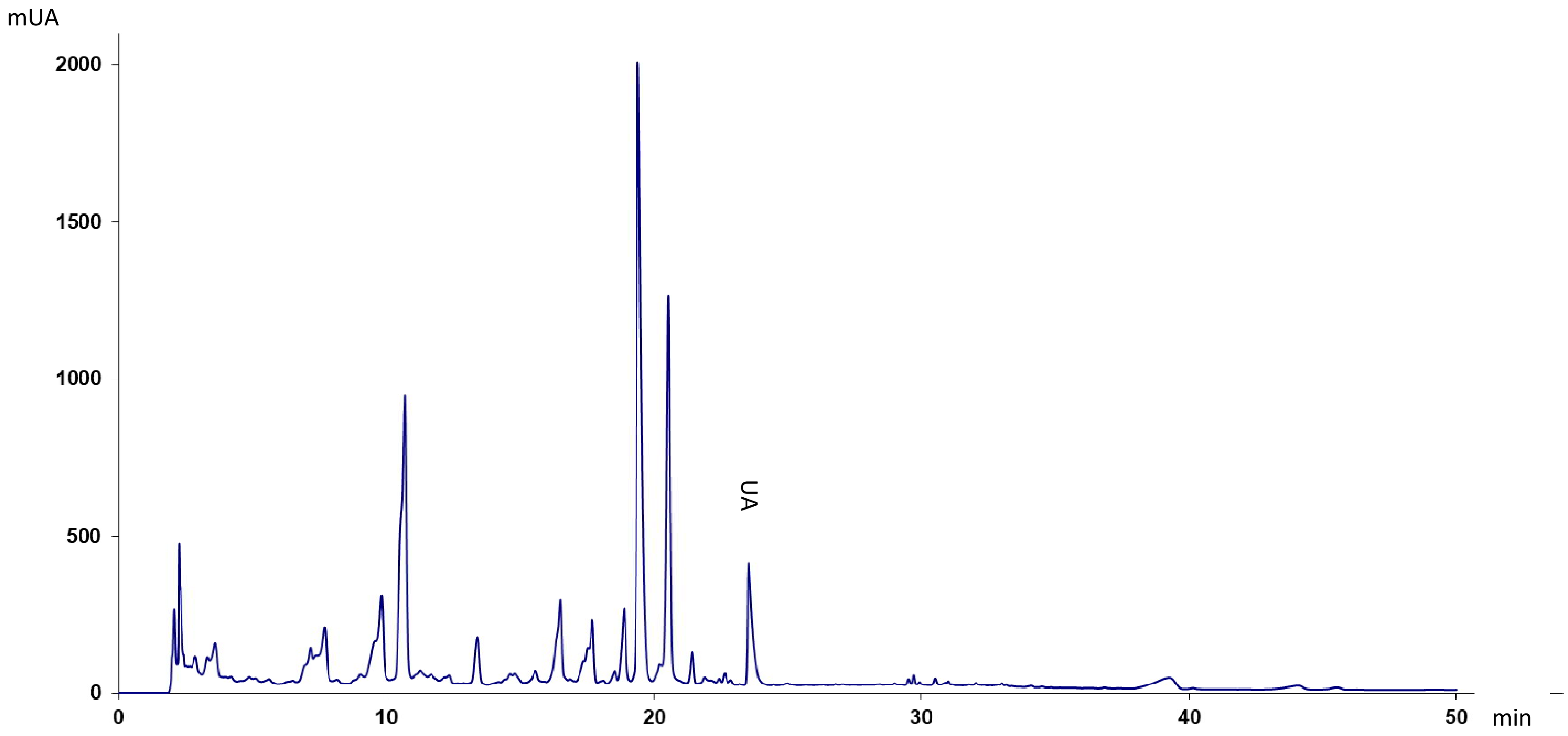

Figure S6. The chromatogram of *Hypogymnia physodes* (HP1) methanolic extract, UA – usnic acid.

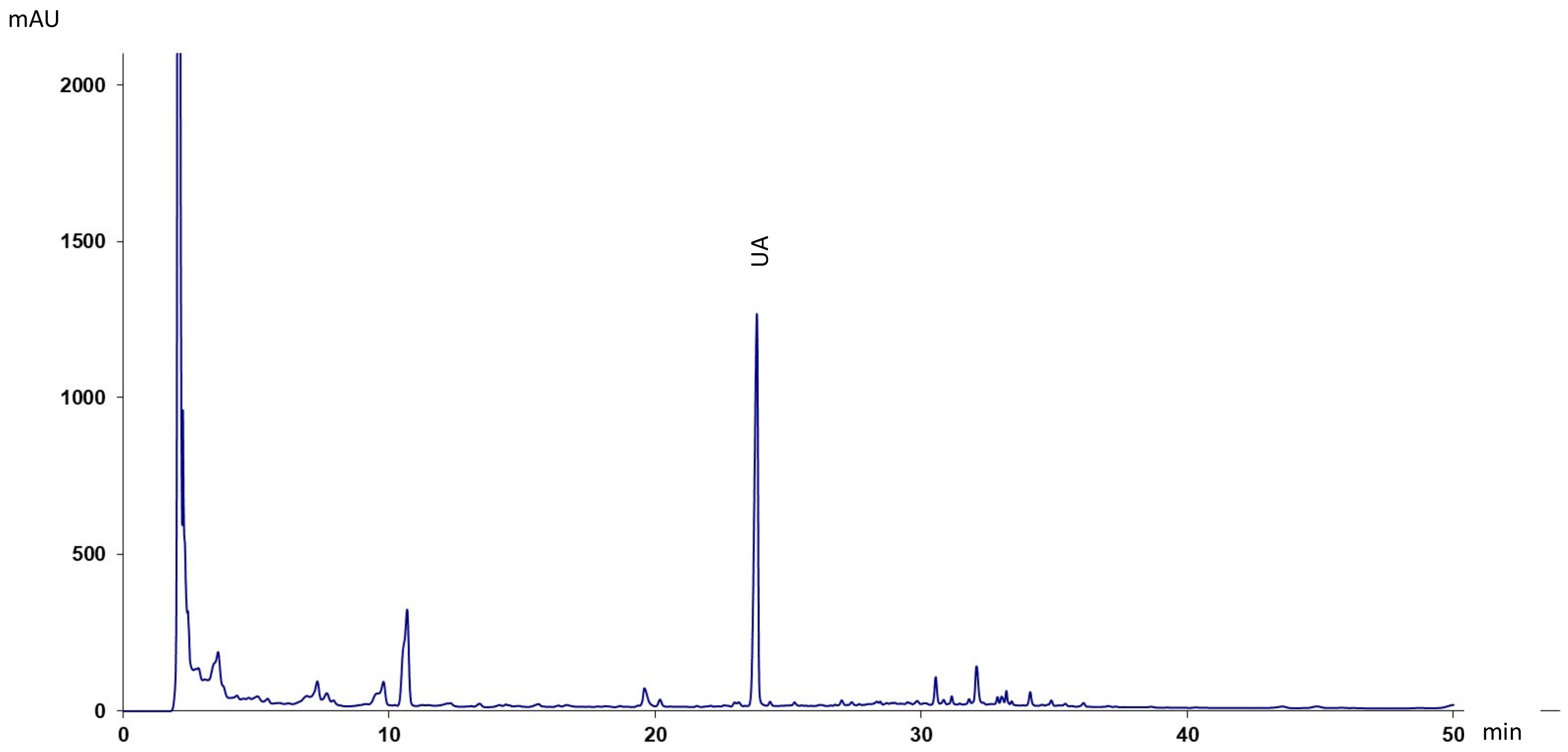

Figure S7. The chromatogram of *Xanthoria parietina* (XP) methanolic extract, UA – usnic acid.
